# A novel riboswitch classification based on imbalanced sequences achieved by machine learning

**DOI:** 10.1371/journal.pcbi.1007760

**Published:** 2020-07-20

**Authors:** Solomon Shiferaw Beyene, Tianyi Ling, Blagoj Ristevski, Ming Chen

**Affiliations:** 1 Department of Bioinformatics, College of Life Sciences, Zhejiang University, Hangzhou, China; 2 School of Medicine, Zhejiang University, Hangzhou, Zhejiang, China; 3 Faculty of Information and Communication Technologies, Bitola, St. Kliment Ohridski University Bitola, ul. Partizanska Bitola, Republic of North Macedonia; University of Ottawa, CANADA

## Abstract

Riboswitch, a part of regulatory mRNA (50–250nt in length), has two main classes: aptamer and expression platform. One of the main challenges raised during the classification of riboswitch is imbalanced data. That is a circumstance in which the records of a sequences of one group are very small compared to the others. Such circumstances lead classifier to ignore minority group and emphasize on majority ones, which results in a skewed classification. We considered sixteen riboswitch families, to be in accord with recent riboswitch classification work, that contain imbalanced sequences. The sequences were split into training and test set using a newly developed pipeline. From 5460 *k*-mers (*k* value 1 to 6) produced, 156 features were calculated based on *CfsSubsetEval* and *BestFirst* function found in WEKA 3.8. Statistically tested result was significantly difference between balanced and imbalanced sequences (*p* < 0.05). Besides, each algorithm also showed a significant difference in sensitivity, specificity, accuracy, and macro F-score when used in both groups (*p* < 0.05). Several *k*-mers clustered from heat map were discovered to have biological functions and motifs at the different positions like interior loops, terminal loops and helices. They were validated to have a biological function and some are riboswitch motifs. The analysis has discovered the importance of solving the challenges of majority bias analysis and overfitting. Presented results were generalized evaluation of both balanced and imbalanced models, which implies their ability of classifying, to classify novel riboswitches. The Python source code is available at https://github.com/Seasonsling/riboswitch.

## Author summary

Machine learning application has been used in many ways in bioinformatics and computational biology. Its use in riboswitch classification is still limited. Existing attempts showed challenges due to imbalanced sequences. Algorithms can classify sequences with majority and minority groups, but they tend to ignore minority group and emphasize on majority class, consequential return a skewed classification. We used a new pipeline including SMOTE for balancing sequences that showed better-classified riboswitch as well as improved performance of algorithms selected. Statistically significant difference observed between balanced and imbalanced in sensitivity, specificity, accuracy and F-score, this proved balanced sequences better for classification of riboswitch. Biological functions and motif search of *k*-mers in riboswitch families revealed their presence in interior loops, terminal loops and helices. Some of the *k*-mers were reported to be riboswitch motifs of aptamer domains and critical for metabolite binding. The pipeline can be used in machine learning and deep learning study in other domains of bioinformatics and computational biology suffering from imbalanced sequences. Finally, scientific community can use python source code, the work done and flow to develop packages.

This is a *PLOS Computational Biology* Methods paper.

## Introduction

Riboswitches, primarily discovered in bacteria [1], are parts of regulatory noncoding mRNA [2]. Riboswitches are predominantly present in the 5’ untranslated region [3,4]. They have complex folded structure [5,6]. They act as a switch to transform the transcription or translation of the genes. In transcription, they turn a downstream gene ‘off’ or ‘on’ [7] in changing concentration of specific metabolites or ligands [8] and allow microbes to quickly react to change degrees of metabolites [7]. A high-throughput platform showed how RNA makes structural transitions [9] kinetically compete during transcription in a new mechanism for riboswitch.

A riboswitch (50–250 nt in length) has two main classes aptamer and an expression platform [10]. The aptamer region is a highly conserved domain, which is a site for binding of ligands (metabolites) and the latter one alters conformation on the binding of metabolite and hence regulates the expression of related genes [5,6]. Recently, almost over twenty diverse classes of riboswitches have been found in bacteria, archaea [11,12] and eukaryote. The majority of the riboswitch classes are in bacteria [12,13]. Thiamine pyrophosphate (TPP) is the only eukaryotic riboswitch. It is detected in Arabidopsis thaliana. TPP was also found in some fungi [13] for instance *Neurospora crassa*, in algae [14,15].

The last two decades have revealed incredible advancement in big and complex omics data due to emerged novel high-throughput experimental technologies such as next-generation sequencing [16,17]. Numerous bioinformatics databases are available to gather data for riboswitches analyses and assemble the information regarding diverse functionality of RNA molecules [18], including GenBank, National Center for Biotechnology Information (NCBI), Rfam [19], Protein Data Bank (PDB), RiboD [20] and European Bioinformatics Institute (EMBL-EBI).

Many efforts have been made to develop suitable bioinformatics tools to predict the presence of riboswitches in ribonucleic acid sequences [18]. The most commonly used computation tools for the analysis of riboswitches are: RiboD [20], Riboswitch finder [21], RibEx [22], RiboSW [23], mFold [24] and RegRNA [18]. These available bioinformatics tools use Covariance Model (CM), Support Vector Machine (SVM) and Hidden Markov Model (HMM) algorithm. Most research exists mainly depending on the principal of multiple sequence alignment to investigate conserved sequences in already reported riboswitch. The attempt was to find out the conserved sequence of previously reported riboswitches in a targeted manner. Most reported studies depend on multiple sequence and thus limited for the classes of riboswitches in a family [21–24]. However, research conducted on frequency-dependent revealed its importance in the classification of riboswitch [25,26]. Frequency-dependent classification uses *k*-mers counts. *K*-mers counts have many application like, building de Bruijn graphs [27] in case of *de novo* assembly from very big number of short reads, generated from next-generation sequencing (NGS), used in case of multiple sequence alignment [28], and repeat detection [29].

A tremendous amount of data are generated every day that create the demand for learning algorithms that can classify, predict and analyze data more accurately [30]. There are two classification categories: classification of binary format [31] and multi-class classification [32,33].

The concept of imbalanced sequences is defined as follows. Each family in classes of riboswitch with majority groups has more than two thousand class and minority group below thousands, which is considered as an imbalanced sequence. Whereas, the imbalanced group used and treated with Synthetic Minority Over-Sampling Technique (SMOTE) and thereafter it is called a balanced sequences. The classification with imbalanced data gives favors for a sample with the majority class [30]. Classifiers trained by balanced sequences are defined as balanced classifiers. Imbalanced data occur as a circumstance where the records of a sequences of one class are very little in relation to the other classes’ sequences. This leads classifier algorithms to ignore minority groups and emphasize on majority class, which can result in skewed accuracy of the classifier. The value of the accuracy of the classifier might be high, but minority class misclassified. Several findings have been done for riboswitch classification [25,26] based on imbalanced data. However, data resampling can be a solution to handle the class imbalance problems [30]. Synthetic Minority Over-Sampling Technique (SMOTE) has been discovered in 2002, which is a sampling-based algorithm. Synthetic Minority Over-Sampling Technique [34] balances the class distribution of imbalanced sequences through an incrementing approach on some virtual samples.

To address the needs for riboswitch prediction, nucleotide frequency counts are considered. SMOTE was used for resampling. Different machine learning algorithms are used for evaluation such as: Random forest (RF) randomizes the variables (columns) and data (rows), generating thousands of classification trees, and then summarizing the results of the classification tree [35]. Gradient boosting (GB) is a boosting algorithm, which belongs to ensemble learning as well as random forest and proved to have great performance in imbalance problem. It builds the model in a stage-wise fashion, and generalizes them by allowing optimization of an arbitrary differentiable loss function. Support vector machine (SVM) is a simple and efficient method for solving the quadratic programming problem through computing the maximum marginal hyper-plane. In SVM, the kernel function implicitly defines the feature space for linear partitioning, which means the choice of kernel function is the largest variable of SVM [35]. K-Nearest Neighbors (KNN) is classifier offers numerous choices to speed up the undertaking to locate nearest neighbors, Naïve Bayes (NB) classifier based on Bayes' theorem [25]. This is a probability-based model in Bayesian networks. Multilayer perceptron (MLP) is a commonly used machine learning algorithm. It is a deep, artificial neural network. A neural network is comprised of layers of nodes which activate at various levels depending on the previous layer’s nodes [25]. The performances of each algorithm on classification were derived from the confusion matrix, which reveals the number of matches correctly and mismatched instances of riboswitches. Specificity, sensitivity, accuracy, and macro F-score were calculated. That parameters are the main performance evaluation criteria for machine learning algorithms [35–38].

## Results

### Sequences preprocessing and feature selection

**Fig 1:**
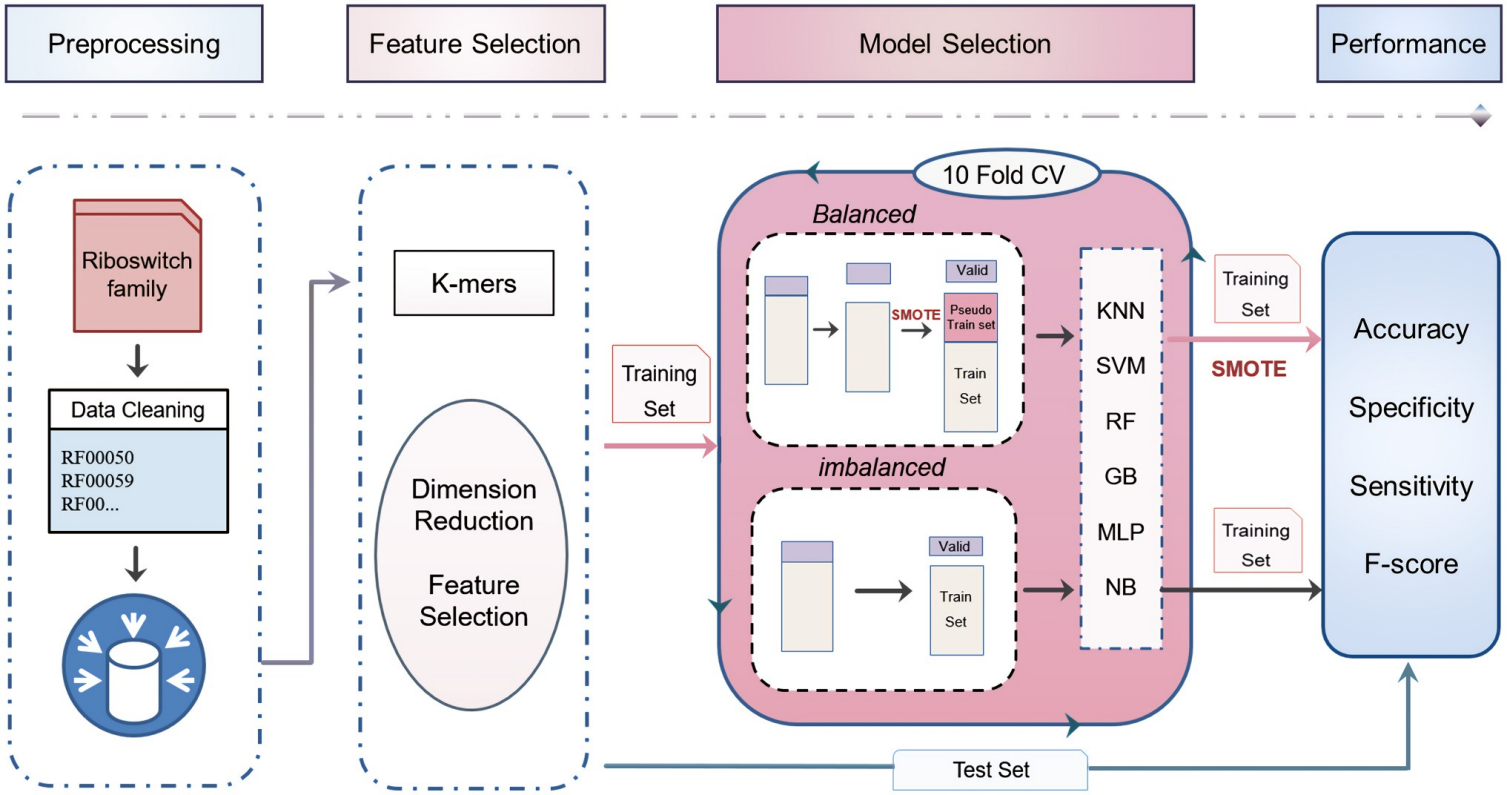
The workflow used to analyze imbalanced and balanced sequences. It was used to compare the computational performance of machine learning algorithms for classification.

**Fig 2:**
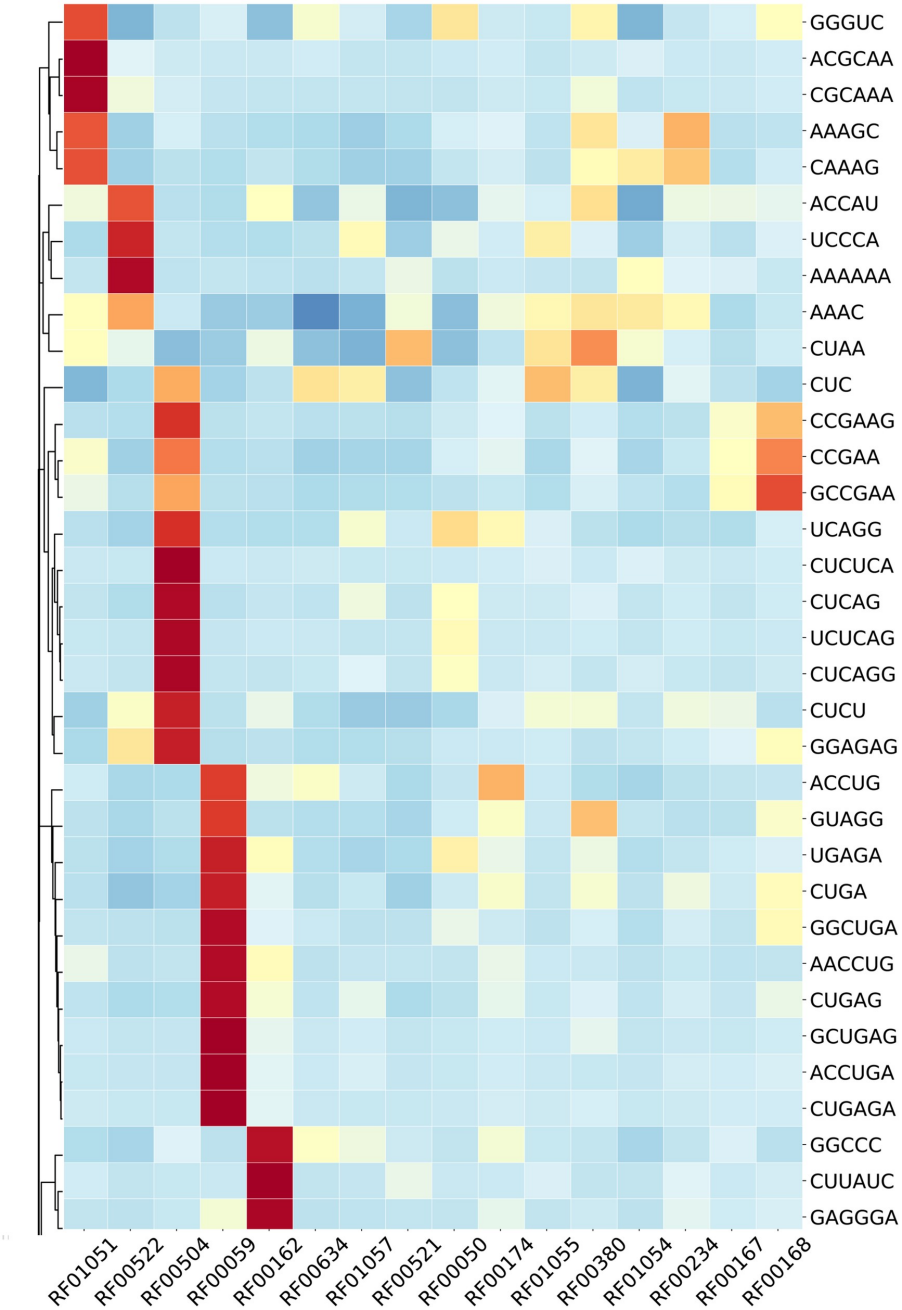
Heat-map in this figure represented as row-normalized *k*-mer counting distribution. Rows correspond to the *k*-mers, and columns revealed 16 families of riboswitch. The clustering heatmap depicts feature clustering, clustered features were essential for classification in that family. Red means a high relatively counting number while blue means lower (see details in S1 Fig).

**Fig 3:**
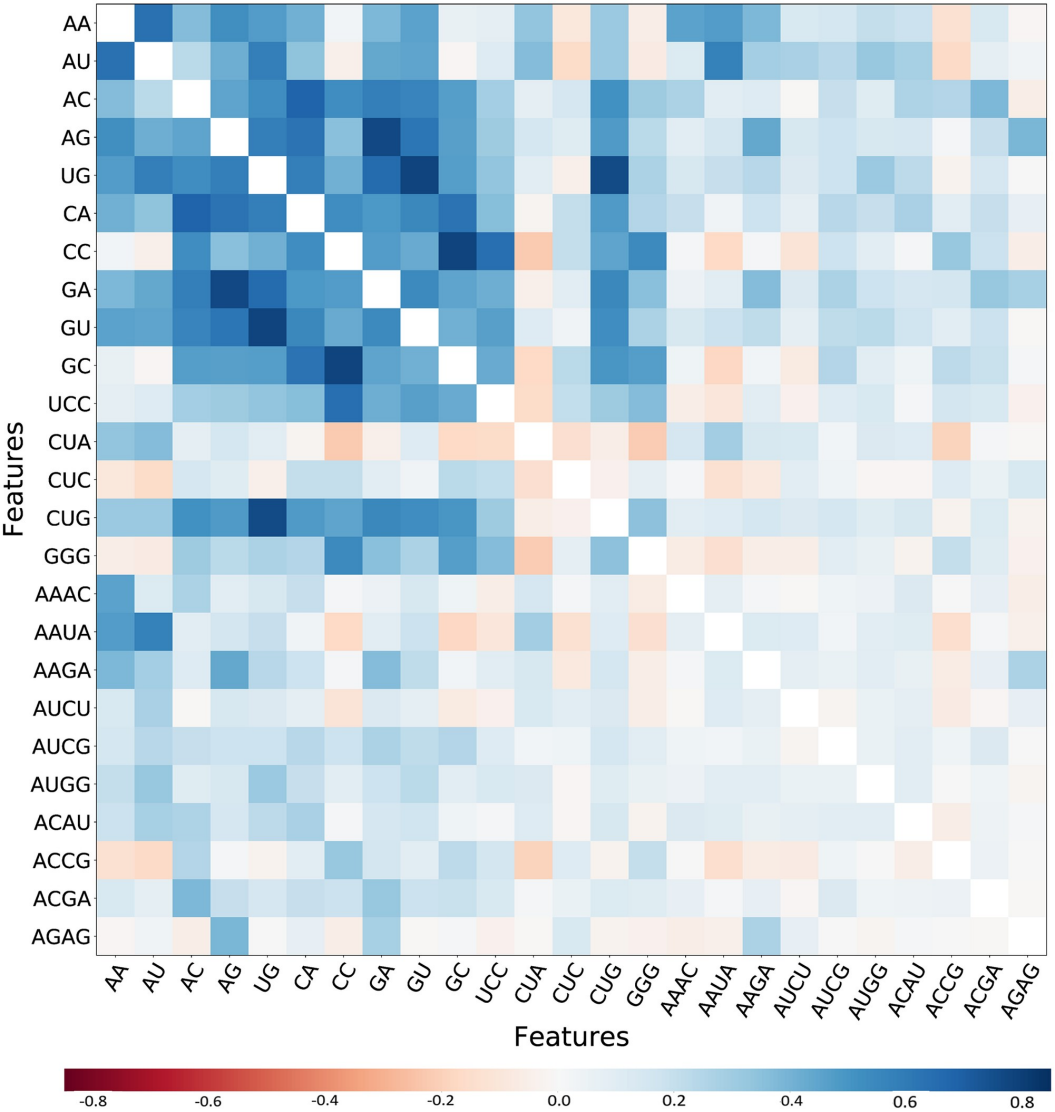
Heat-map showed features correlation. It depicts the diagonal white line represented their correlation factor equals to one. Blue means a positive correlation, while red means a negative correlation (see details in S2 Fig).

Riboswitch families considered for this analysis and their corresponding details were presented and analyzed in Fig 1 and features where clustered in Fig 2 (see detail in S1 Fig). Looking into instances in riboswitch, there were differences in representation between families range in distribution from Cobalamin riboswitch (4,826 sequence classes) to PreQ1-II (39 sequence classes). Out of 16 riboswitch class, Cobalamin riboswitch, TPP riboswitch (THI element), and Glycine riboswitch contributed for 68% and the remaining 13 riboswitch family has 32% instances. The performances of algorithms and methods were computed and evaluated based on training and test set (details in the methodological approach part). We produced 5460 *k*-mers (*1≤k≤6*) by R script and exported a matrix containing all riboswitch sequences and their corresponding *k*-mers value. A Sequences preprocessing and feature selection afterward, 156 features were calculated based on the Correlation-based Feature Subset Selection algorithm (*CfsSubsetEval*) and Best First Search (*BestFirst*) in WEKA 3.8 [39] (Fig 3 and detail in S2 Fig), which was consistent with previous research [26].

### Imbalanced class on classification performance

**Fig 4:**
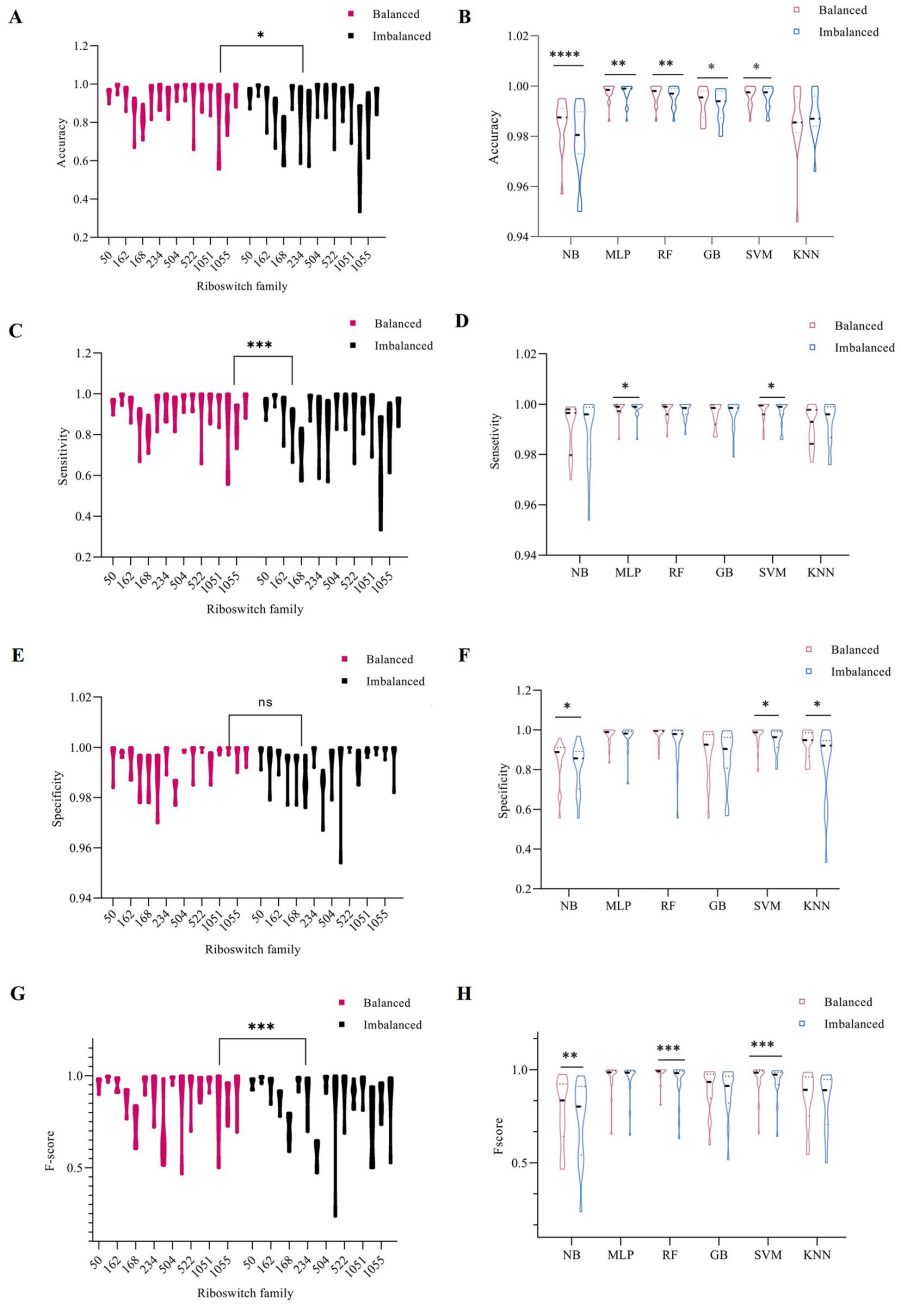
The figures showed a comparison of the balanced and imbalanced sequences and performance of classifiers. It has been done using the Wilcoxon rank test, A) Accuracy showed significant difference between balanced and imbalanced sequences (*p* < 0.05) C) Sensitivity showed very significant difference between balanced and imbalanced sequences (*p* < 0.001) E) Specificity revealed no significant differences at all levels G) F-score showed very significant difference between balanced and imbalanced sequences (*p* < 0.001). Classifiers performance evaluation on imbalanced and imbalanced sequences shown as B) Accuracy resulted to have significant difference in all classifiers except KNN (*p* < 0.05, *p <* 0.01, *p <* 0.001) D) Sensitivity observed to have significant difference in only MLP and SVM (*p* < 0.05) whereas the remaining algorithms showed no differences F) Specificity depicted significant differences in NB, SVM and KNN (*p* < 0.05) on the other hand MLP, RF and GB showed no differences in both sequences group H) F-score depicted very significance differences in NB (*p* < 0.01), RF (*p* < 0.001) and SVM (*p* < 0.001) whereas KNN and MLP showed no differences. Violin box was used to depict the statistical differences between two group were provided as the plots. (* indicated significant difference of *p* < 0.05, ** denoted very significant difference of *p* < 0.01, and *** showed very significant difference *p* < 0.001).

After feature selection, sequences containing 156 *k*-mers values were split into 70% training dataset and 30% test dataset. Improved cross-validation method in training dataset was used to validate both imbalanced models and balanced models, while the remaining test set was applied to test generalizations of those models. All the following results are results based on the test set, which can demonstrate their ability to classify novel sequences. Classifiers on minority class resulted in F-score value from 0.50 (NB) to 0.94 (MLP), while on majority class, the range is from 0.91 to 1.00, as indicated in Table 1 and Fig 4. Riboswitch families considered for classification were present in S1 Table. The average performance of each classifier is computed using mean and standard deviation for parameters: accuracy, specificity, sensitivity and macro F-score.

**Table 1:** Accuracy, sensitivity, specificity and F-score. This parameters were used for Naïve Bayes(NB), Multilayer Perceptron(MLP), Random Forest(RF), Gradient Boosting(GB), Support Vector Machine(SVM) and K-Nearest Neighbors(KNN) algorithms evaluation when applied on the imbalanced sequences. The color trend of F-score from blue to red indicates performance from the best to the poorest. Accuracy, sensitivity, specificity, and F-score are represented in the table as Acc, Sen, Spec, and F-sco, respectively.

	NB				MLP				RF				GB				SVM				KNN			
Family	Acc	Sen	Spec	F-sco	Acc	Sen	Spec	F-sco	Acc	Sen	Spec	F-sco	Acc	Sen	Spec	F-sco	Acc	Sen	Spec	F-sco	Acc	Sen	Spec	F-sco
RF00521	0.95	0.91	0.95	0.24	1.00	0.97	1.00	0.97	1.00	1.00	1.00	1.00	1.00	0.82	1.00	0.89	1.00	0.97	1.00	0.99	1.00	0.94	1.00	0.94
RF00522	1.00	0.66	1.00	0.69	1.00	1.00	1.00	0.99	1.00	1.00	1.00	1.00	1.00	0.83	1.00	0.89	1.00	1.00	1.00	0.99	1.00	1.00	1.00	0.97
RF00059	0.98	0.94	1.00	0.97	1.00	1.00	1.00	1.00	1.00	1.00	1.00	0.99	1.00	1.00	1.00	0.99	1.00	0.99	1.00	0.99	0.98	0.98	0.98	0.96
RF00174	0.95	0.87	0.99	0.91	1.00	0.99	1.00	0.99	0.99	1.00	0.99	0.99	0.99	0.99	0.98	0.98	0.99	0.99	0.99	0.98	0.97	0.94	0.98	0.94
RF00504	0.97	0.83	1.00	0.90	1.00	1.00	1.00	1.00	1.00	1.00	1.00	0.99	0.98	0.99	0.98	0.93	1.00	1.00	1.00	1.00	0.99	0.95	0.99	0.96
RF01051	0.98	0.69	1.00	0.81	1.00	0.99	1.00	0.99	1.00	1.00	1.00	0.99	0.99	0.94	1.00	0.96	1.00	0.99	1.00	0.98	0.99	0.95	1.00	0.95
RF01057	0.98	0.88	0.98	0.53	1.00	0.98	1.00	0.96	1.00	0.98	1.00	0.99	1.00	0.92	1.00	0.96	1.00	0.96	1.00	0.97	1.00	0.84	1.00	0.87
RF00050	0.99	0.87	1.00	0.92	1.00	0.97	1.00	0.98	1.00	0.98	1.00	0.98	0.99	0.93	1.00	0.96	1.00	0.96	1.00	0.98	0.99	0.94	0.99	0.92
RF00162	0.98	0.74	1.00	0.84	1.00	0.99	1.00	0.99	1.00	0.98	1.00	0.98	0.99	0.95	1.00	0.97	1.00	0.98	1.00	0.99	0.99	0.94	0.99	0.91
RF00234	0.99	0.89	0.99	0.79	1.00	0.99	1.00	0.99	1.00	0.96	1.00	0.98	1.00	0.97	1.00	0.98	1.00	0.84	1.00	0.91	0.99	0.59	1.00	0.70
RF00634	0.99	0.89	1.00	0.38	1.00	0.98	1.00	0.98	0.99	0.95	0.99	0.98	0.98	0.80	0.99	0.87	0.99	0.97	0.99	0.97	0.98	0.90	0.99	0.84
RF01055	0.99	0.85	1.00	0.81	1.00	0.96	1.00	0.96	1.00	0.87	1.00	0.93	0.99	0.73	1.00	0.83	1.00	0.93	1.00	0.95	0.99	0.61	1.00	0.74
RF00380	0.97	0.97	0.97	0.62	0.99	0.97	0.99	0.96	0.99	0.86	0.99	0.92	0.99	0.57	0.99	0.84	0.99	0.92	0.99	0.95	0.98	0.83	0.99	0.82
RF00167	0.98	0.66	0.98	0.78	0.99	0.93	1.00	0.91	0.99	0.91	1.00	0.91	0.99	0.87	1.00	0.88	0.99	0.91	1.00	0.92	0.98	0.86	1.00	0.84
RF00168	0.97	0.83	0.98	0.59	0.99	0.73	1.00	0.77	0.99	0.77	1.00	0.78	0.99	0.60	1.00	0.68	0.99	0.80	1.00	0.80	0.99	0.57	1.00	0.66
RF01054	1.00	0.56	1.00	0.50	1.00	0.89	1.00	0.94	1.00	0.56	1.00	0.71	1.00	0.89	1.00	0.80	1.00	0.89	1.00	0.94	1.00	0.33	1.00	0.50

**Fig 5:**
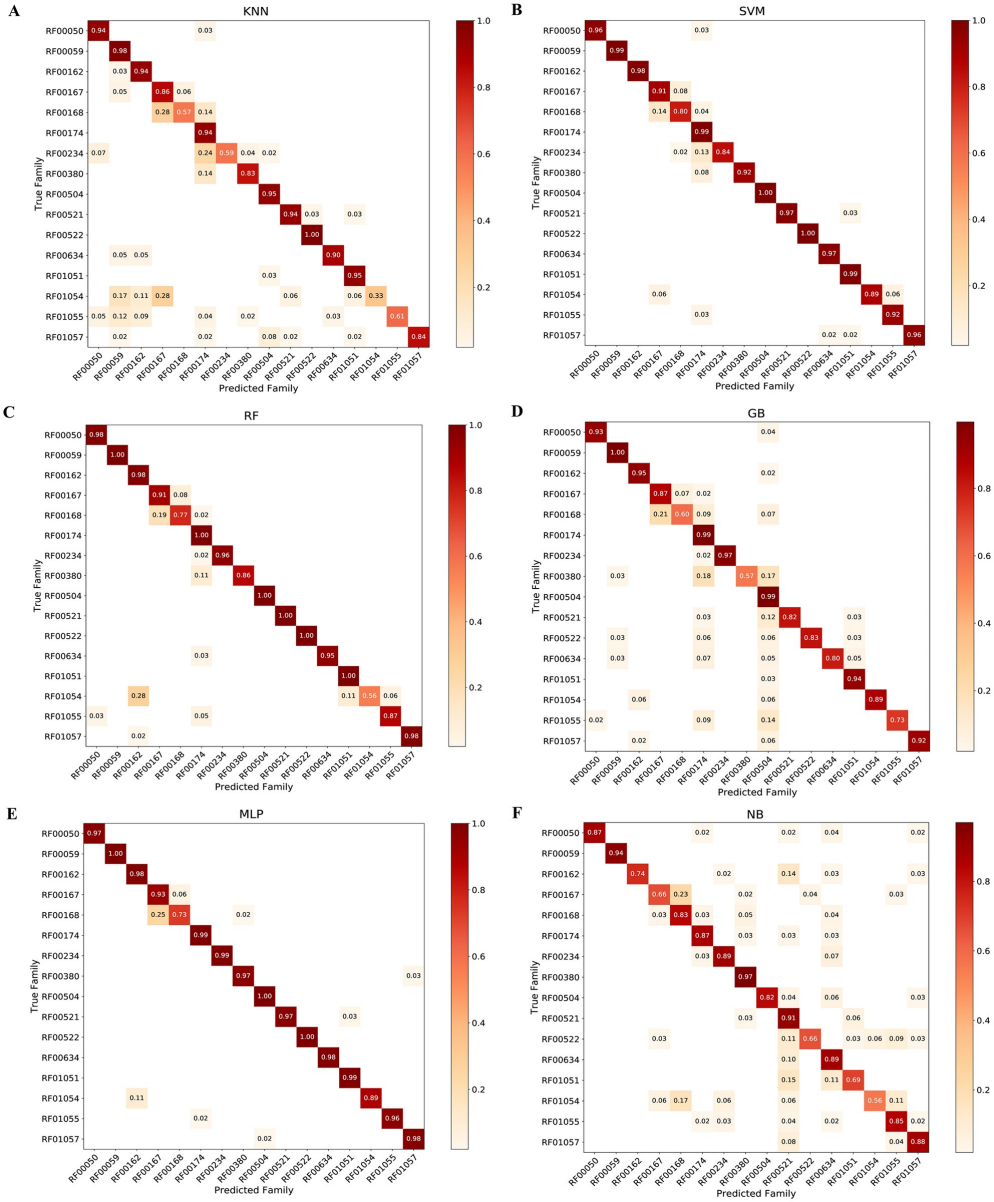
Confusion matrix for imbalanced sequences from independent test experiments depicted true family and predicted family. For the classifiers such as: A) K-Nearest Neighbors, B) Support Vector Machine, C) Random Forest, D) Gradient Boosting, E) Multilayer Perceptron and F) Naïve Bayes.

The comparative analysis of six algorithms has revealed that MLP performs best, while NB performed the poorest results (S2 Table). RF00174, RF00059, RF00504, RF00522 classified better than others with minority classes like RF01054, RF00634, RF00380 (Table 1). F-scores of MLP and RF for the majority group (RF00174) were 0.997 and 0.996, respectively. In the minority group, classifiers with high accuracy had F-score up to 0.50 in the case of NB. The computed minimum value in overall NB analysis in RF01054, RF00634, and RF00521 were 0.50, 0.38, and 0.24, respectively. Accuracy of all algorithms across all riboswitch families showed values greater than 0.97. In confusion matrix, predicted family and true family exhibited performance of classifiers and riboswitch classification (Fig 5).

### SMOTE balancing on classifiers performance

**Fig 6:**
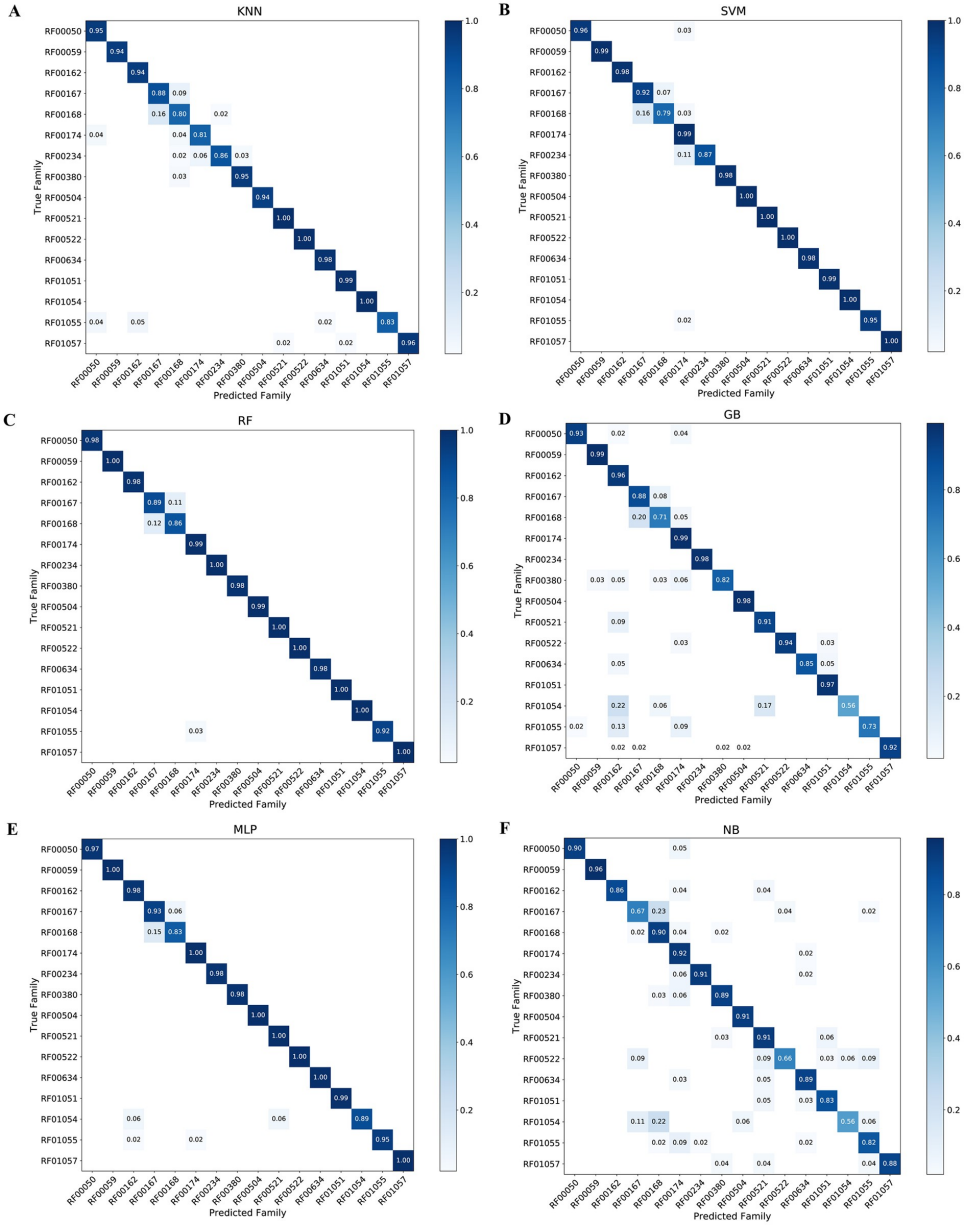
Confusion matrix for the balanced sequences from independent test experiments. It showed True family and Predicted value with classifiers as: A) K-Nearest Neighbors, B) Support Vector Machine, C) Random Forest, D) Gradient Boosting, E) Multilayer Perceptron and F) Naïve Bayes.

The overall analysis computed for frequency counts of all families had discovered improved performances of classifiers (S2 Table, Table 2 and Fig 4). RF00059 and RF00174 results showed F-score between 0.93 and 1.00. In the case of NB and KNN, results of the F-score indicated their poorer performance with a value less than 0.84. Performance evaluations have revealed that KNN, NB, SVM, MLP, RF and GB can be used for classification of riboswitch (Fig 6).

**Table 2:** Performances of Naïve Bayes (NB), Multilayer Perceptron (MLP), Random Forest (RF), Gradient Boosting (GB), Support Vector Machine (SVM) and K- Nearest Neighbors (KNN). These algorithms were evaluated using the balanced sequences from 16 riboswitch families measured by using accuracy, sensitivity, specificity and F-score. The color trend of F-score from blue to red indicates performance from the best to the poorest. Accuracy, sensitivity, specificity, and F-score are represented in the table as Acc, Sen, Spec, and F-sco, respectively.

	NB				MLP				RF				GB				SVM				KNN			
Family	Acc	Sen	Spec	F-sco	Acc	Sen	Spec	F-sco	Acc	Sen	Spec	F-sco	Acc	Sen	Spec	F-sco	Acc	Sen	Spec	F-sco	Acc	Sen	Spec	F-sco
RF00059	0.99	0.96	1.00	0.98	1.00	1.00	1.00	1.00	1.00	1.00	1.00	1.00	1.00	0.99	1.00	0.99	1.00	0.99	1.00	0.99	0.98	0.94	1.00	0.96
RF00234	1.00	0.92	1.00	0.88	1.00	0.98	1.00	0.99	1.00	1.00	1.00	1.00	1.00	0.98	1.00	0.98	1.00	0.87	1.00	0.92	0.99	0.86	0.99	0.72
RF00521	0.98	0.91	0.99	0.47	1.00	1.00	1.00	0.99	1.00	1.00	1.00	1.00	1.00	0.91	1.00	0.89	1.00	1.00	1.00	1.00	1.00	1.00	1.00	0.96
RF00522	1.00	0.66	1.00	0.70	1.00	1.00	1.00	1.00	1.00	1.00	1.00	1.00	1.00	0.94	1.00	0.97	1.00	1.00	1.00	0.99	1.00	1.00	1.00	0.99
RF01054	1.00	0.56	1.00	0.50	1.00	0.89	1.00	0.94	1.00	1.00	1.00	1.00	1.00	0.56	1.00	0.71	1.00	1.00	1.00	1.00	1.00	1.00	1.00	0.84
RF01057	0.99	0.88	0.99	0.69	1.00	1.00	1.00	0.99	1.00	1.00	1.00	1.00	1.00	0.92	1.00	0.96	1.00	1.00	1.00	0.99	1.00	0.96	1.00	0.82
RF00162	0.99	0.86	1.00	0.91	1.00	0.98	1.00	0.98	1.00	0.99	1.00	0.99	0.98	0.96	0.99	0.91	1.00	0.99	1.00	0.99	0.98	0.94	0.99	0.91
RF00174	0.96	0.92	0.97	0.93	1.00	1.00	1.00	0.99	1.00	0.99	1.00	0.99	0.99	0.99	0.99	0.98	0.99	0.99	0.99	0.98	0.95	0.81	1.00	0.89
RF00504	0.99	0.91	1.00	0.95	1.00	1.00	1.00	0.99	1.00	0.99	1.00	0.99	1.00	0.98	1.00	0.99	1.00	1.00	1.00	0.99	0.99	0.94	1.00	0.96
RF01051	0.99	0.83	1.00	0.91	1.00	0.99	1.00	0.99	1.00	1.00	1.00	0.99	1.00	0.97	1.00	0.97	1.00	0.99	1.00	0.99	1.00	0.99	1.00	0.97
RF00050	0.99	0.90	1.00	0.94	1.00	0.97	1.00	0.98	1.00	0.97	1.00	0.98	0.99	0.93	1.00	0.96	1.00	0.96	1.00	0.98	0.98	0.95	0.98	0.90
RF00380	0.98	0.89	0.98	0.69	0.99	0.99	0.99	0.96	0.99	0.94	0.99	0.98	0.99	0.82	0.99	0.84	0.99	0.99	0.99	0.98	0.98	0.95	0.98	0.73
RF00634	0.99	0.89	1.00	0.64	1.00	1.00	1.00	0.99	1.00	1.00	1.00	0.98	0.98	0.85	0.99	0.87	0.99	0.98	0.99	0.99	0.99	0.98	0.99	0.86
RF01055	0.99	0.82	0.99	0.79	1.00	0.95	1.00	0.95	1.00	0.93	1.00	0.96	1.00	0.73	1.00	0.85	1.00	0.95	1.00	0.96	0.99	0.83	0.99	0.73
RF00167	0.98	0.67	0.98	0.77	0.99	0.93	1.00	0.93	0.99	0.89	0.99	0.91	0.99	0.89	1.00	0.88	0.99	0.93	1.00	0.93	0.99	0.88	0.98	0.85
RF00168	0.98	0.90	0.98	0.62	0.99	0.83	1.00	0.84	0.99	0.85	0.99	0.81	0.99	0.71	1.00	0.72	0.99	0.79	1.00	0.80	0.98	0.80	0.98	0.60

As presented, Random Forest and MLP exhibited the consistently higher accuracy and F-score values compared to NB, GB, SVM and KNN. Fig 4 and Table 2 have shown that SMOTE improves riboswitch classification and algorithm performances.

The overall accuracy of classifiers trained with SMOTE analyzed sequences (balanced sequences) showed consistent and better results than with imbalanced sequences (S2 Table and Tables 1 and 2). The specificity of NB, MLP, RF, GB, SVM and KNN was better in the balanced classifiers than imbalanced sequences ones. Calculated sensitivity results were slightly better in balanced instances. Surprisingly, evidence discovered in that F-score value in all the models showed that balanced training sequences could improve the classification of riboswitches. When tested by independent test sequences, balanced sequences trained classifiers increased not only classification accuracy, but also algorithms performances than control groups. Balanced sequences increased not only classification accuracy but also algorithms performances. Table 2 has depicted F-score values increasing from 0.50 while in the case of the imbalanced sequences to 0.84.

### Application of statistical significances

Statistical computation using the Wilcoxon rank test [39] between balanced and imbalanced sequences depicts significant differences between these two groups. In addition, the performance of NB, MLP, RF, GB, SVM and KNN statistically showed variation in accuracy, specificity, sensitivity and F-score values. Statistically very significant differences were noticed between balanced and imbalanced in F-score and sensitivity (*p <* 0.001) and accuracies were significantly different (*p* <0.05), whereas specificity showed no significant difference between the two groups (Fig 4, S3 Table).

SVM was a very significant difference in all parameters used for performance evaluation, F-score (*p* < 0.001) whereas accuracy, specificity and sensitivity were significantly different (*p* < 0.05). RF performance in both groups has shown very significant differences in F-score (*p* < 0.001) and accuracy (*p* < 0.01) (Fig 4 and S2 Table). In KNN we did not notice statistical significant differences in all used parameters, except significant differences in specificity (*p* < 0.05).

MLP of the balanced and imbalanced group depicted very significant differences in accuracy and sensitivity (*p* < 0.01). GB showed significant differences only in accuracy (*p* < 0.05). Finally, both imbalanced and balanced sequences in the case of NB have shown very significantly differences in F-score (*p* < 0.01), accuracy (*p* < 0.001), whereas specificity was a significant difference (*p* < 0.05). Accuracy of all classifiers is significantly different at different levels in both groups except in KNN (Fig 4 and S3 Table).

### Biological functions of clustered *k-mer*s

**Fig 7:**
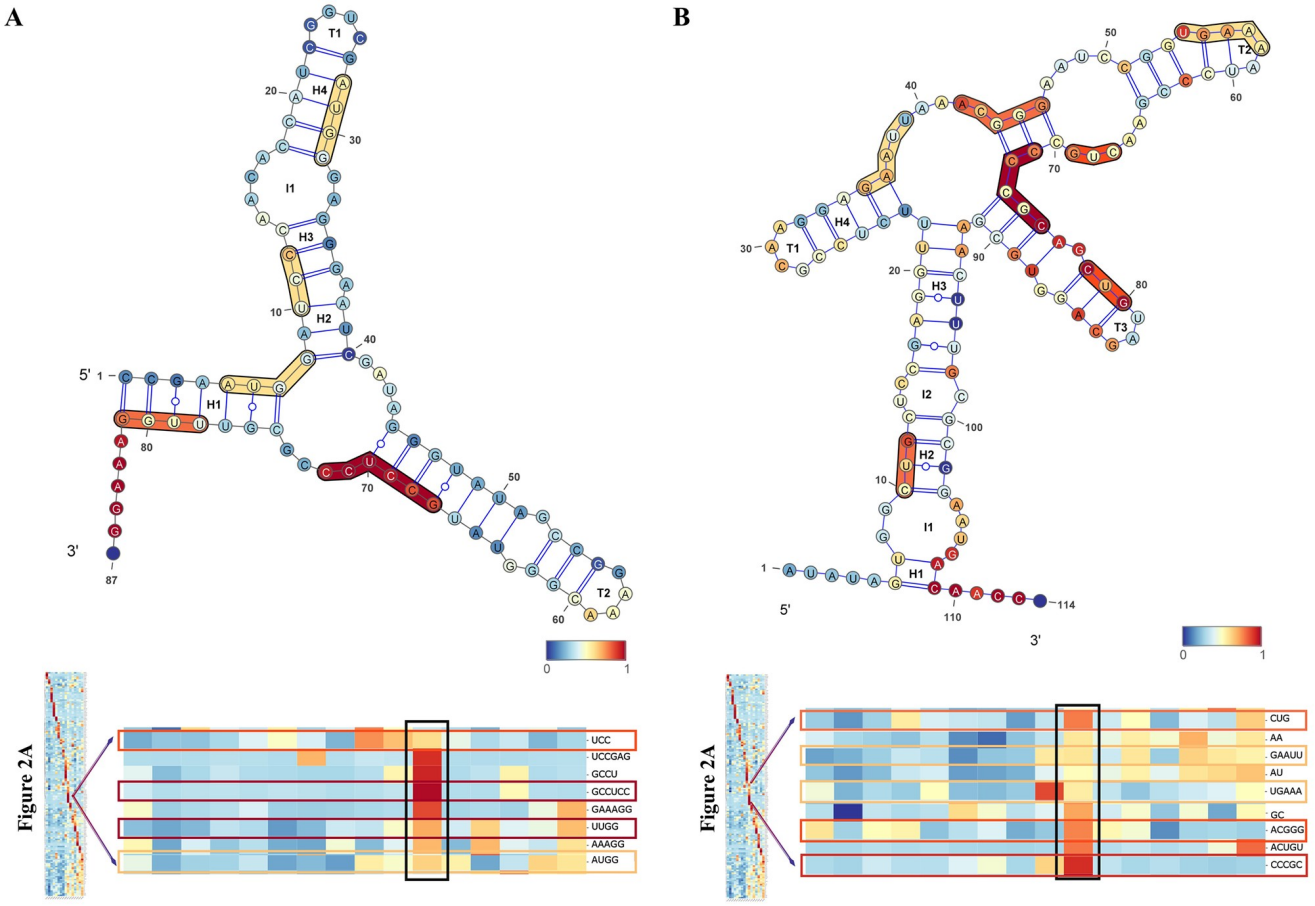
Secondary structure of RF00174 Cobalamin riboswitch (Acido bacterium) (A) and RF01055 MOCO riboswitch class (B). In every individual base, the color gradient scale represents a normalized hit number from 156 features aligned to the sequence. The different color scale in each region represents its coverage of the *k*-mers in the family that it represents. Whereas, I, H, and T are abbreviations for Interior loops, Helices, and Terminal loops, respectively.

*K-mer*s counting was extracted from distribution heat-map (Fig 2, S1 Fig), which depicted feature clustering and high relative count number. These clustered *k-mer*s were used for biological function and motif searching. Accordingly, in Table 3 riboswitch families and their *k-mer*s were used to verify their biological functions. Structural analysis from *k-mer*s coverage results is depicted in the case of RF00174 (A) and RF01055 (B). In every individual base, the color gradient scale indicates a normalized count. Results depict different color scale in each region and their interior loops, helices, and terminal loops (Fig 7).

**Table 3:** Clustered *k-mers* from S1 Fig used for validation of their biological function and reported riboswitch motifs. Nucleotide location designated refers to match with their position reported in reference.

Rfam ID	*K-mer*s	Position	Riboswitch function and motifs	Ref
**RF00168**	UCAU	U57-U60	Motifs predicted to interact with the Nova-1 protein	[40]
**RF01051**	CAAAG	C22-G26	Secondary structure representation of the crystallized c-di-GMP aptamer, necessary for c-di-GMP binding pocket formation	[41]
	GGUC	G8-C11	Found in Helices P1 area beginning of 5′UTR	[41]
**RF00522**	AAAAAA AAAC	A27-A31 A30-C33	Overlaying *K-mers* in the 3′ aptamer domain, rich in A, which has unique folding pseudoknot that compresses PreQ1	[42]
	UCCCA	U24-A18	Found in P2 of preQ1 riboswitch aptamer structure	[42]
**RF00504**	CCGAAG	C168-G173	The glycine-mediated changes in spontaneous cleavage (GAA)	[43]
	CUCU	C204-U207	In glycine riboswitch, secondary structure and in-line decreasing cleavage pattern	[43]
**RF00059**	UGAGA	U39-A43	The pyrimidine part of TPP is bound by bulge J3-2 located in the pyrimidine-sensor helix P2-P3	[44]
**RF00162**	GAGGGA	G19-A24	It is a kink-turn motif that allows pseudoknot interaction. It interacts with SAM which helps to make stable formation, can cause the downstream expression platform to form a rho-independent TT (transcriptional terminator), turning off gene expression	[45]
**RF00634**	CAACC CCCUC	C54-C58 C57-C61	Overlapping *k-mers* in SAM-IV RNA binds SAM, last C in cleavage increased by SAM	[46]
**RF01057**	AGGCUC	A61-C66	In P1 SAH riboswitch control reporter gene Expression, *ahcY* 5'UTR	[47]
	CGCU	C28-U31	In SAH riboswitch hairpin loops of P4	[47]
**RF00521**	GCUAAA	G42-A47	Secondary structure of the Env12 metX SAM-II riboswitch its base-pairing reflecting the tertiary structure of the SAM-bound RNA	[48]
**RF00050**	AGUC	A126-C129	In the secondary structure of FMN, the first three AGU make hairpin loops and identified from B. cleavage.	[49]
	ACAGU GGCGGU	A137-U141 G56-U61	Form Secondary-structure model of the 165 ribD RNA and side hairpin between P2 and 165 ribD RNA and side hairpin	[49]
**RF00174**	CCCGC AGUCAG	C70-C74	Predicted secondary structure of the cobalamin riboswitch in the btuB leader region of *Synechococcus* sp. Strain. The boxed bases represent the B12 box-P1 helix interface, where a CC-to-TT (UU in the RNA structure	[50]
**RF01055**	GAAAGG	G120-G125	Containing AGG at the site of Ribosomal Binding Site (RBS) located at multiple junction site. Region of the central multi-stem junction in Sequence of the 138 moaA Moco RNA.	[51]
	GCCU	G18-U21	Found in a Moco RNA at left multiple junction site	[51]
	GCCUCC	G106-C111	In Moco RNA, the last UCC makes parts of multiple junctions in P4.	[51]
**RF00380**	UGAGG	U28-G32	*k-mers* that found in part of a conserved bulge-stem region of Secondary structure of the 5`M-box portion	[52]
**RF01054**	AAAGG	A83-G87	Structural modulation and nucleotides comprise a conserved Shine–Dalgarno (SD)	[53]
	AGCAU	A58-U62	Unpaired Structural modulation containing constant cleavage	[53]
	AGAAAA	A88-A93	Structural modulation, AGAA in decreasing cleavage and AA in constant cleavage	[53]
**RF00234**	AGCGC	A12-C16	Downstream of the ribozyme cleavage site Ribozyme core site P2a	[54]
	ACGAGG	A53-G56	Ribozyme core region (Unpaired)	
**RF00167**	CUAC	C50-C53	structural features of the guanine aptamer domain and critical for metabolite binding	[55]

## Discussion

Machine learning has an enormous capacity to boost our knowledge in the classification of riboswitch, an area that is still in the early stage of a comprehensive investigation. Numerous machine learning applications have been developed based on different methods to detect riboswitch. However, most riboswitch classification studies applied machine learning algorithms on the imbalanced sequences [25,26]. Several findings revealed the impact of imbalance sequences on correct classification and performance of algorithms [25,26,30]. Chawla and colleagues proposed SMOTE method of treating imbalanced sequences for better classification of majority and minority instances [30,34]. SMOTE based balancing of sequences improves the oversampling minority classes accurately and also produces sequences that do not influence majority class.

In this analysis, there are imbalances of instances in the riboswitch family. Comparative results revealed the reality of the impact of such an imbalance on classification which has widely been reported (S1 Table and Table 1). Imbalanced distribution exhibited variation from 4826 majority class (Cobalamin riboswitch) to 39 minority class (PreQ1-II riboswitch). General classifiers, when encountering such imbalanced data, favor class with majority instances [30,34]. The analysis also revealed in imbalanced and balanced confusion matrix the same problem (Figs 5 and 6). Out of 16 riboswitch class, cobalamin riboswitch, TPP riboswitch (THI element), and glycine riboswitch sum up contribution was 68% while the remaining 13 riboswitch family has 32% instances. In Table 2, full sequences grouped into two sets training (70%) and test set (30%) was selected and performances of classifiers were evaluated regarding sensitivity, accuracy, specificity and F-score. The correlation heat-map in Fig 3 (see detail in S2 Fig) indicates the relationships between *k*-mers.

Imbalanced sequences in riboswitch showed different performances of classifiers ranked as: MLP—the best and NB—the poorest regarding their mean scores that range from 0.771 to 0.961. In Table 2, individual score results of this method have shown best result in RF00234, RF00522, RF01057 (1.00 in RF): greater values than reported in other study using BLAST^+^ [26,56], which is most popular tools in analysis of sequence similarity [56] and others [25,26]. Conversion of sequences into vector revealed good results in both groups used for analysis (S2 Table and Tables 1 and 2). In protein study, protein sequence converted into feature vectors showed good performance in cases of SVM and KNN [57–60]. RF00174, RF00059, RF00504, RF00522 predicted better than others with minority classes like RF01054, RF00634, RF00380 (Tables 1 and 2). The class with maximum instances (RF00174) resulted in an F-score value greater than 0.94 in all classifiers except NB, which had a value less than 0.93 in both cases.

NB classifier depicted poor performance in imbalanced sequences compared to other classifiers. Its accuracy, sensitivity, specificity, and F-score had the following values 0.979, 0.989, 0.814 and 0.705, respectively (S2 Table). These results were improved to 0.985, 0.991, 0.841 and 0.771 when sequences were balanced. Compared with the F-score value reported by Hugo and colleagues (NB-HEXCFS- 0.525), the changes indicated the influence of imbalanced sequences on the performance of classifiers. Similarly, improved performance of NB on large sequences has been reported [61].

Table 2, S2 Table, and Fig 4 indicate that the proposed method of balancing instances increases classifier performances. The used approach was also reported as a solution for machine learning [62]. RF shows the best result followed by MLP, which revealed optimal performance. On the other hand, Naïve Bayes has poor performances in imbalanced sequences classification, which is in accordance with Mwagha and colleagues [63,64]. The overall comparison revealed that balanced classifiers are better for classification of riboswitch, their performances were compared to BLAST^+^ [26] and other finding (S3 Table and Tables 1 and 2).

The *k*-mers position in the secondary structure illustrated riboswitch biological function and motif (Table 3 and Fig 7). In RF00174, *CCCGC k*-mers had predicted the secondary structure of the cobalamin riboswitch in the btuB leader region of Synechococcus. In cases like RF00168, *UCAU k*-mer had motifs predicted to interact with the Nova-1 protein, overlaying *K*-mers in the 3′ aptamer domain, rich in A, which has unique folding pseudoknot that compresses PreQ1 [40]. Turning off gene expressing observed in RF00162 with *GAGGGA k*-mer, is a kink-turn motif which allows pseudoknot interaction. It interacts with SAM which helps to make stable formation, and can cause the downstream expression platform to form a rho-independent TT (transcriptional terminator), turning off gene expression [45]. Overall, *k*-mers and their biological function for this study are summarized and described in Table 3.

The pipeline can be used in machine learning and deep learning study in other domains of bioinformatics and computational biology that suffer from imbalanced sequences. Finally, the scientific community can use the python source code for analysis of interest as well as to develop suitable software packages.

## Methodology

We showed a complete evaluation of different machine learning approaches for classification and predicting regulatory riboswitches. First of all, we present the benchmark sequences and data mining approach followed by feature engineering that was done through testing. Besides, model selection methods were used to model and compare balanced and imbalanced sequences problem, as well as determine the best combination of hyperparameters for each classifier [65]. These methods are implemented in an open-source machine learning platform called WEKA 3.8 [66,67], SMOTE [31] and Python 3 [68], which allow evaluating different parameters and algorithms for classification and prediction of the riboswitch. Lastly, we described the results of classifications from the learned models. The workflow for the analysis of imbalanced and balanced sequences used for performance evaluation of different machine learning algorithms found in Fig 1. This workflow can be used for other research areas that suffer from challenges of imbalanced sequences. The python source codes are available at https://github.com/Seasonsling/riboswitch.

### Data preprocessing

Sequences for investigation were collected from Rfam 13.0 [19] and other sequences that were already produced [26], intended for comparison of our new methods. Rfam is a source that collects RNA families including riboswitch [19]. There is a need to use a machine learning approach to train algorithms to classify riboswitch as it has been happening in other areas of bioinformatics. Only 16 families have been used to compare with previous research work and they clearly show the impact of imbalanced training sequences on the performance of classifiers. Preprocessing, cleaning and filtering were done, as well as handling missing values, noisy data, redundant features and irrelevant features to affect the accuracy of the model [67]. The sequences that contain sequences per family are shown in S1 Table.

### Feature selection

FASTA format sequences were used for *k*-mer (*1 ≤ k ≤ n)* frequency counts through executing in the R package called *kcount* [69]. In order to obtain a sufficiently informative *k*-mer counting matrix for the task [70], we set *k* value to 6 and finally got 5,460 features. This *k-mers* composition was used to make frequencies of each riboswitch. This avoids unnecessary computing power consumption and dimensional disaster caused by extremely sparse matrices due to high *k* values as well.

Attribute evaluators *CfsSubsetEval* and *BestFirst* were used for dimensionality reduction and searching of the space of attribute subsets by greedy hill-climbing augmented with a backtracking facility [71], which was consistent with some other researchers [26]. WEKA 3.8 was used to implement the task [66,67]. Feature selection was done for the dimensionality reduction and thus for decrease processing load [72,73].

### Imbalanced data

The sequences for this finding contains the imbalanced sequences ranging from 4,826 instances (RF00174) to 39 (RF01051) instances (S1 Table). Learning from the imbalanced sequences that become critical concerns nowadays, particularly when minority class contains small instances in its sequences [25,26,74]. Mainstream methods of dealing with imbalance data can be roughly divided into two categories. The first category considers the difference in the cost of different misclassifications [75], while the second one mainly focuses on training data sampling strategies. Here over-sampling and under-sampling were conventional techniques used to adjust class distribution. However, traditional random oversampling adopts the strategy of merely copying samples to increase the minority samples, which is prone to the problem of overfitting that makes the information learned by the model over-fitted and not generalized [76].

SMOTE improved scheme based on random oversampling was applied [59]. The basic idea of the SMOTE algorithm is to analyze a small number of samples and to add new samples to the data set based on a small number of samples.

The used algorithm flow is as follows:

For each sample *x* in a few classes, calculate the distance from all samples in a few samples sets by Euclidean distance, and get its *k*-nearest neighbors.

Set a sampling ratio according to the sample imbalance ratio to determine the sampling magnification *N*. For each minority sample *x*, randomly select several samples from its *k*-nearest neighbors, assuming that the selected neighbor is $\hat{x}$.

For each randomly selected neighbor $\hat{x}$, construct a new sample with the original sample according to the following formula:

$$x_{ne\omega }=x+rand\left(0,1\right)\left(\hat{x}-x\right)$$ (1)

SMOTE was deployed through importing “imblearn.over_sampling” module in Python 3 and it was applied both in the corresponding training set of 10-fold cross-validation and building final model processes, as shown in Fig 1.

### Machine learning models

A crucial step in machine learning is model selection, as the performance of algorithms is sensitive to the calibration parameters. Configuration and choice of the hyper-parameters are found to be crucial. For our data, we calibrated a model using 10-fold cross-validation. Firstly, the complete feature selection of *k-mers* sequences was divided into two parts randomly: 70% of data were training set, while 30% of data as the test set. The 70% training set was used to build multiclass classification models and determine the hyper-parameters through 10-fold cross-validation. Then, the test set was used to test the final generalization performance of the balanced and imbalanced models. In order to increase the credibility of comparison results and to ensure the repeatability of the results, all sequences were chosen randomly. Input data and model parameters except for the step of SMOTE processing were strictly consistent for both balanced and imbalanced models. This task was left to make *pipeline* module and *Pipeline* object in Python package *imblearn* (0.5.0), which ensures that in cross-validation or generalization testing, SMOTE only treats the training data used to build the cross-validation model or the final model. By this means, the validation set in each fold cross-validation was consistent in all models just as in the case of the 30% test set.

During the model selection process, for each algorithm, the grid search method was applied to traverse all hyper-parameter combinations, while 10-fold cross-validation method for evaluating each parameter combination. Specifically, the program randomly divided the 70% training set into ten straight sections. During each cycle of the model training step, nine of those sections were treated by SMOTE (the control group not), and then for model training. Subsequent that, the remaining section of the training set will test the model and obtain a series of test indicators, including macro F-score, macro recall and macro precision. The valid score was calculated through the below formula:

$$Score_{valid}=F-score_{macro}*0.6+recall_{macro}*0.2+precision_{macro}*0.2$$ (2)

Running the above cycle ten times independently, we take the average of ten valid scores as the overall performance index of the model under this parameter combination. After evaluating all the parameter combinations with the grid-search method, we pick the model with the highest comprehensive performance index as the final model.

### Experimentation classifiers

Random Forest is a commonly used machine learning algorithm [77] with different successful function in computing and bioinformatics [77–79]. It randomizes the variables (columns) and data (rows), generating thousands of classification trees, and then summarizing the results of the classification tree. In this research, the mean decrease impurity method was used.

SVM is a simple and efficient method for solving the quadratic programming problem [80] through computing the maximum marginal hyper-plane [66]. In SVM, the kernel function implicitly defines the feature space for linear partitioning, which means the choice of kernel function is the largest variable of SVM.

Gradient boosting is a boosting algorithm, which belongs to ensemble learning as well as random forest and proved to have great performance in imbalance problem. It builds the model in a stage-wise fashion, and generalizes them by allowing optimization of an arbitrary differentiable loss function [81].

Another classifier is *k*-Nearest Neighbors (KNN) which also named IBK (instant-based learning with parameter *k*). This classifier offers numerous choices to speed up the undertaking to locate nearest neighbors [67], NB (Naïve Bayes) classifier based on Bayes' theorem [49]. This is a probability-based model in Bayesian networks [82]. MLP is another commonly used machine learning algorithms [83]. ncRNA classification and prediction problems have been widely conducted based on the six selected algorithms for this analysis [84–86] and riboswitch classification and prediction [3,26].

The tuning of KNN, SVM, RF, GB and MLP was carried out on the training set by evaluating the macro F-score in Python 3. The configurations of their parameters are as follows:

KNN: number of *k* = *{2*, *4*, *6*, *8*, *10*, *12*, *14*, *16}*
SVM: type of kernel function = *{linear*, *poly*, *rbf*, *sigmoid}*
RF: with the method of GridSearchCV and kfold = *10*, the number of trees in the forest = *{500*, *1000*, *2000}*, the maximum depth of the tree = *{10*, *15*, *20}*
GB: with the method of GridSearchCV and kfold = *10*, the number of trees in the forest = *{500*, *1000*, *2000}*, learning rate = *{0*.*01*, *0*.*1*, *0*.*05}*, the maximum depth of the tree = *{7*, *9*, *11*, *15}*
MLP: with the method of GridSearchCV and kfold *= 10*, hidden layer size = *{{80*, *80*, *80}*, *{100*, *100*, *100}*, *{150*, *150*, *150}}*, *L2* penalty (regularization term) parameter = *{1e-3*, *1e-4}*, the solver for weight optimization = *{‘adam’*, *‘sgd’}*, tolerance for the optimization = *{1e-8*, *1e-7*, *1e-6}*
Gaussian NB: portion of the largest variance of all features that is added to variances for calculation stability = *{1e-16*, *1e-14*, *1e-12}*

### Evaluation

In order to evaluate the performance of the classifiers, the confusion matrices were used to compute sensitivity, specificity, accuracy and F-score [32,87]. Most researchers used a weighted F-score to evaluate the classifier’s overall performance. However, it leads to assessment bias between majority families and minority families. In this evaluation, we used macro F1 instead, which gives an arithmetic mean of the per-class F1-scores and avoids assessment bias to some extent. A statistical test was carried out in GraphPad Prism 8.3.0 using the Wilcoxon rank test and multiple Wilcoxon rank test at *p* < 0.05, 0.01, 0.001 level (“Wilcoxon rank test were performed using GraphPad Prism version 8.3.0 for Windows, GraphPad Software, La Jolla California USA, www.graphpad.com”).

We used the following abbreviations: True Positives (*TP*), False Positive (*FP*), True Negative (*TN)*, and False Negative (*FN*). The used formulas are as follows:

$$Sensitivity=\frac{TP}{TP+FP}$$ (3)

$$Specificity=\frac{TN}{TP+FN}$$ (4)

$$Accuracy=\frac{TP+TN}{TP+FN+TN+FP}$$ (5)

$$F-score=\frac{2TP}{2TP+FP+FN}$$ (6)

## Supporting information

S1 Table The table used for the purpose of comparison of imbalanced and balanced sequences from Rfam database. The training (70%) and test sequences (30%) for classification and evaluation performance of machine learning algorithms. Feature distribution across different 16 riboswitch families using heat-map is shown in Fig 2.
(DOCX)

S2 Table Classifiers’ performances with balanced and imbalanced sequences arranged in F-score decreasing order in case of the balanced sequences. For a specific classifier, mean represents average sensitivity, specificity, accuracy and F-score value, while standard deviation (SD) depicted variation in different riboswitch families.
(DOCX)

S3 Table The statistical difference of four measurements between the balanced and imbalanced sequences. Bolded *p*-values indicate the statistical difference (SD).
(DOCX)

S1 Fig Heat-map in this figure represented as row and columns. A) row-normalized k-mer counting distribution, rows correspond to the k-mers, and columns revealed 16 families of riboswitch and B) the clustering heatmap depicts feature clustering, clustered features were essential for classification in that family. Red means a high relatively counting number while blue means lower.
(TIF)

S2 Fig Heat-map showed 156 features correlation. The diagonal white line represented their correlation factor equals to one. Blue means a positive correlation, while red means a negative correlation.
(TIF)
