# Supplementary material for: A novel riboswitch classification based on imbalanced sequences achieved by machine learning
Source: PLoS Comput Biol. 2020 Jul 20;16(7):e1007760. doi: 10.1371/journal.pcbi.1007760 (PMC7392346; doi:10.1371/journal.pcbi.1007760)
Supplement: S1 Table — The training (70%) and test sequences (30%) for classification and evaluation performance of machine learning algorithms. Feature distribution across different 16 riboswitch families using heat-map is shown in Fig 2. (DOCX) [file pcbi.1007760.s001.docx]

| **Type of Riboswitch family** | **Rfam ID** | **Full sequences** | **Training Set** | **Test set** |
| --- | --- | --- | --- | --- |
| Purine Riboswitch | RF00167 | 813 | 587 | 226 |
| TPP riboswitch (THI element) | RF00059 | 3616 | 2551 | 1065 |
| SAM riboswitch (S box leader) | RF00162 | 1241 | 852 | 389 |
| Molybdenum Cofactor riboswitch | RF01055 | 303 | 210 | 93 |
| Glycine Riboswitch | RF00504 | 2125 | 1491 | 634 |
| Lysine Riboswitch | RF00168 | 341 | 245 | 96 |
| FMN Riboswitch | RF00050 | 1211 | 827 | 384 |
| Cobalamin riboswitch | RF00174 | 4286 | 2993 | 1293 |
| glmS glucosamine-6-phosphate activated ribozyme | RF00234 | 275 | 181 | 94 |
| SAM riboswitch (alpha-proteobacteria | RF00521 | 167 | 133 | 34 |
| yKoK leader | RF00380 | 217 | 152 | 65 |
| S-adenosyl methionine (SAM) riboswitch | RF00634 | 216 | 155 | 61 |
| PreQ1 Riboswitch | RF00522 | 116 | 81 | 35 |
| Cyclic di-GMP-I riboswitch(c-di-GMP-I) | RF01051 | 1020 | 701 | 319 |
| PreQ1-II (pre queuosine) riboswitch | RF01054 | 39 | 21 | 18 |
| S-adenosyl-L-homocysteine riboswitch | RF01057 | 198 | 148 | 50 |
