# Supplementary material for: A novel riboswitch classification based on imbalanced sequences achieved by machine learning
Source: PLoS Comput Biol. 2020 Jul 20;16(7):e1007760. doi: 10.1371/journal.pcbi.1007760 (PMC7392346; doi:10.1371/journal.pcbi.1007760)
Supplement: S2 Table — For a specific classifier, mean represents average sensitivity, specificity, accuracy and F-score value, while standard deviation (SD) depicted variation in different riboswitch families. (DOCX) [file pcbi.1007760.s002.docx]

|  | **Accuracy** | | **Specificity** | | **Sensitivity** | | **F-score** | |
| --- | --- | --- | --- | --- | --- | --- | --- | --- |
| **Model** | Imbalanced | Balanced | Imbalanced | Balanced | Imbalanced | Balanced | Imbalanced | Balanced |
| **RF** | 0.996±0.004 | 0.997±0.001 | 0.925±0.118 | 0.972±0.011 | 0.997±0.003 | 0.998±0.001 | 0.945±0.083 | 0.975±0.049 |
| **MLP** | 0.997±0.004 | 0.997±0.001 | 0.958±0.068 | 0.969±0.012 | 0.998±0.004 | 0.998±0.001 | 0.961±0.057 | 0.970±0.042 |
| **SVM** | 0.996±0.005 | 0.996±0.001 | 0.943±0.059 | 0.964±0.014 | 0.997±0.005 | 0.997±0.001 | 0.955±0.049 | 0.968±0.050 |
| **GB** | 0.992±0.006 | 0.993±0.001 | 0.864±0.132 | 0.883±0.031 | 0.996±0.006 | 0.996±0.001 | 0.901±0.082 | 0.904±0.089 |
| **KNN** | 0.988±0.009 | 0.986±0.003 | 0.824±0.192 | 0.928±0.017 | 0.993±0.008 | 0.991±0.002 | 0.844±0.134 | 0.856±0.111 |
| **NB** | 0.979±0.013 | 0.985±0.002 | 0.814±0.117 | 0.841±0.028 | 0.989±0.014 | 0.991±0.002 | 0.705±0.212 | 0.771±0.163 |
