# Supplementary material for: A novel riboswitch classification based on imbalanced sequences achieved by machine learning
Source: PLoS Comput Biol. 2020 Jul 20;16(7):e1007760. doi: 10.1371/journal.pcbi.1007760 (PMC7392346; doi:10.1371/journal.pcbi.1007760)
Supplement: S3 Table — Bolded p-values indicate the statistical difference (SD). (DOCX) [file pcbi.1007760.s003.docx]

| **Measurement** | **NB** | **MLP** | **RF** | **GB** | **SVM** | **KNN** |
| --- | --- | --- | --- | --- | --- | --- |
| **Accuracy** | **0.0002** | **0.0096** | **0.0033** | **0.0326** | **0.0203** | 0.8169 |
| **Sensitivity** | **0.0496** | 0.118 | 0.1135 | 0.104 | **0.0173** | **0.0403** |
| **Specificity** | 0.1461 | **0.0038** | 0.3557 | 0.0977 | **0.0161** | 0.9305 |
| **F-score** | **0.0019** | 0.1406 | **0.0002** | 0.131 | **0.0008** | 0.95 |
