# Supplementary figures and images for: A novel riboswitch classification based on imbalanced sequences achieved by machine learning
Source: PLoS Comput Biol. 2020 Jul 20;16(7):e1007760. doi: 10.1371/journal.pcbi.1007760 (PMC7392346; doi:10.1371/journal.pcbi.1007760)

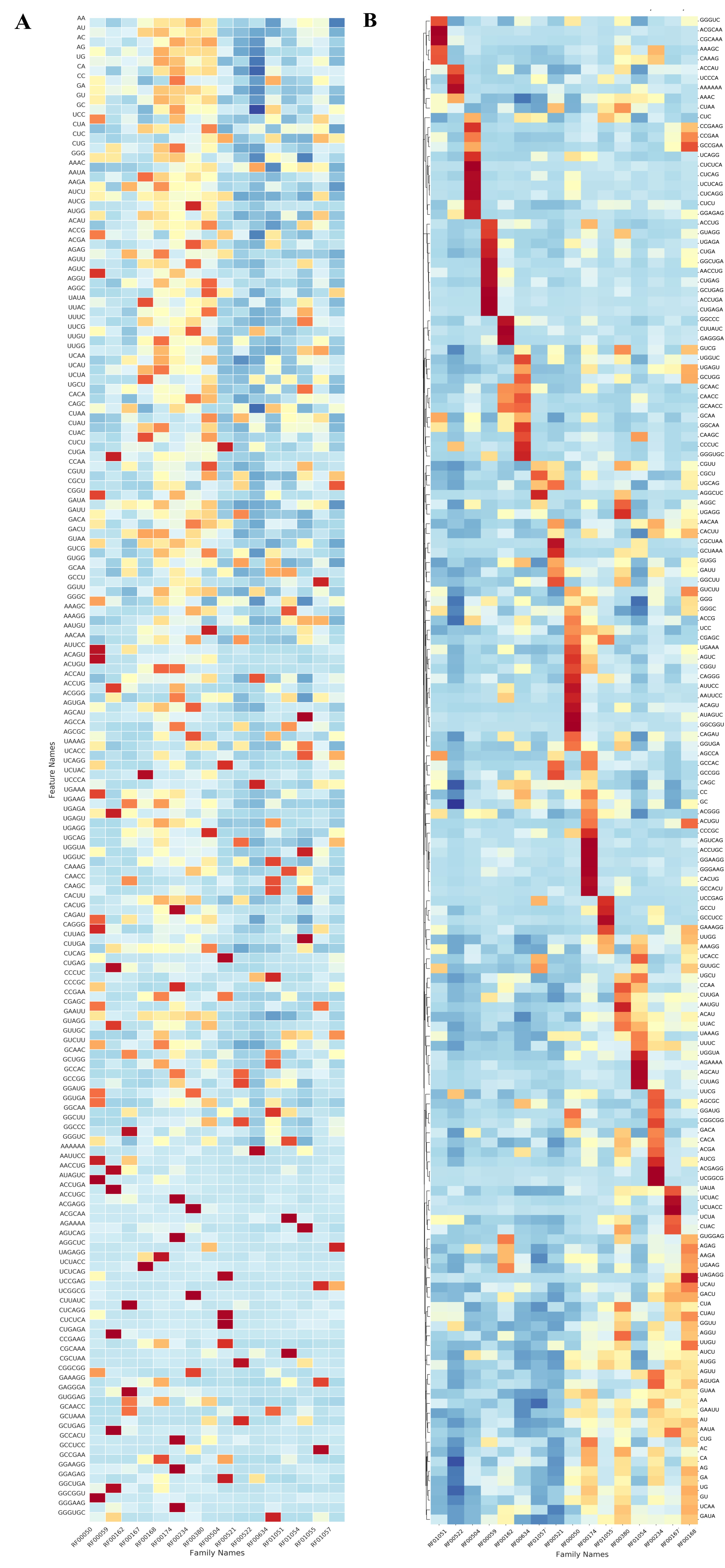

Supplement: S1 Fig — A) row-normalized k-mer counting distribution, rows correspond to the k-mers, and columns revealed 16 families of riboswitch and B) the clustering heatmap depicts feature clustering, clustered features were essential for classification in that family. Red means a high relatively counting number while blue means lower. (TIF) [file pcbi.1007760.s004.tif]

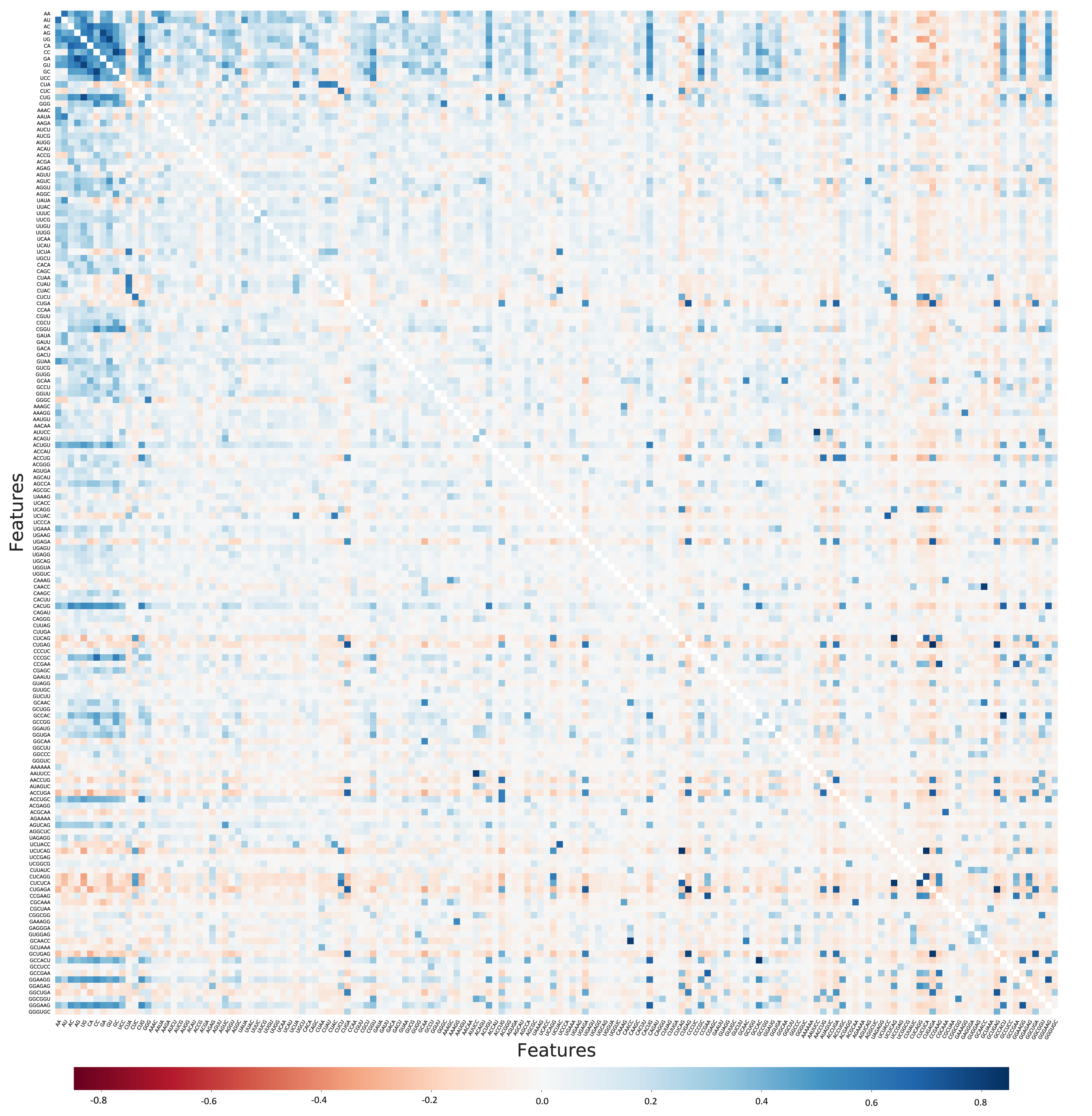

Supplement: S2 Fig — The diagonal white line represented their correlation factor equals to one. Blue means a positive correlation, while red means a negative correlation. (TIF) [file pcbi.1007760.s005.tif]
